# Online mindfulness-based cognitive therapy for people with multiple sclerosis with mild-to-moderate depression– protocol for a type 1 hybrid effectiveness-implementation study

**DOI:** 10.1371/journal.pone.0362553

**Published:** 2026-11-13

**Authors:** Robert Simpson, Jillian Scandiffio, Laura Langer, Sarah Munce, Ashvene Sureshkumar, Anthony Feinstein, Mark Bayley, Caroline Bowman, Tania R. Bruno, Dawn Ehde, Katherine Knox, Nanette Lai, Penelope Smyth, Abhimanyu Sud, Sarah J. Donkers

**Affiliations:** 1 Rehabilitation Sciences Institute, University of Toronto, Toronto, Canada; 2 St. Michael’s Hospital, Unity Health Toronto, Toronto, Canada; 3 Toronto Rehabilitation Institute, University Health Network, Toronto, Canada; 4 Institute of Health Policy, Management, and Evaluation, University of Toronto, Toronto, Canada; 5 Bloorview Research Institute, Holland Bloorview Kids Rehabilitation Hospital, Toronto, Canada; 6 Department of Psychiatry, Temerty Faculty of Medicine, University of Toronto, Toronto, Canada; 7 Department of Psychiatry, Sunnybrook Health Sciences Centre, Toronto, Canada; 8 Department of Family Medicine, McMaster University, Hamilton, Canada; 9 Department of Rehabilitation Medicine, University of Washington, Seattle, United States of America; 10 Department of Physical Medicine and Rehabilitation, College of Medicine, University of Saskatchewan, Saskatoon, Canada; 11 Ontario Veterinary College, University of Guelph, Guelph, Canada; 12 Department of Medicine (Neurology), Faculty of Medicine & Dentistry, University of Alberta, Edmonton, Canada; 13 Department of Family and Community Medicine, University of Toronto, Toronto, Canada; 14 Humber River Health, Toronto, Canada; 15 School of Rehabilitation Science, University of Saskatchewan, Saskatoon, Canada; PLOS: Public Library of Science, UNITED KINGDOM OF GREAT BRITAIN AND NORTHERN IRELAND

## Abstract

**Background:**

Mindfulness-based cognitive therapy (MBCT) is an effective treatment for recurrent depression in the general population and is recommended in clinical guidelines for those with mild-moderate depression. However, the effectiveness of MBCT in people with multiple sclerosis (PwMS) with mild to moderate depression is unknown. Further, how best to tailor and implement MBCT for PwMS is largely unstudied. This manuscript describes the study protocol for a hybrid-effectiveness study to evaluate online MBCT tailored for PwMS.

**Methods:**

This type 1 hybrid effectiveness-implementation study will employ an embedded mixed methods experimental design. Quantitative findings will be collected through a powered waitlist randomized controlled trial examining online MBCT in a minimum of 152 PwMS with case-ness of mild-moderate depression (Clinical Trials Gov #NCT07742579). The intervention will consist of an online 8-week MBCT course with weekly sessions conducted by qualified mindfulness instructors experienced int the care of PwMS. Questionnaires will be collected at baseline, post-intervention, and 3 months later. The primary outcome will be depression, measured using the Hospital Anxiety and Depression Score – Depression (HADS-D). An intention-to-treat analysis will be conducted. Qualitative findings will be collected through semi-structured interviews with 30–40 MBCT participants, HCPs, MBCT instructors, and MS decision makers. Thematic analyses will be used to analyze interview data. The RE-AIM PRISM tool will be used to evaluate implementation and mixed data assessed in a joint display.

**Conclusion:**

The results from this study will provide evidence regarding the effectiveness of online MBCT in PwMS with mild to moderate depression, recommendations for intervention tailoring and implementation. Findings will be disseminated through traditional and novel, creative knowledge mobilization methods.

## Introduction

Multiple sclerosis (MS) is a highly disabling chronic inflammatory neurodegenerative condition which affects over 2,800,000 worldwide [1]. MS affects females at a higher rate than males, with females diagnosed at approximately a 3:1 ratio to males [2]. People living with multiple sclerosis (PwMS) rarely experience the condition as a stand-alone diagnosis. The most common comorbidity is depression which affects ~23.7–30.5% [3,4] PwMS and is typically chronic. The etiology of depression in PwMS is likely multifactorial, including genetic, immune-inflammatory, and psychosocial [5], however comorbid depression is commoner in those PwMS who are aged 42–57, have a secondary progressive phenotype, and are more physically disabled [6]. Comorbid depression strongly correlates positively with fatigue [7] and self-stigma [8], and negatively with self-efficacy and self-esteem [6]. Of added concern, those PwMS with comorbid depression are less likely to adhere to disease modifying therapies (DMTs) for MS [9], and more likely to engage in adverse health behaviours such as smoking and alcohol use [6]. Although comorbid depression exerts strong negative effects on quality of life (QoL) in PwMS [10], it is treatable. Limited evidence supports the use of pharmacological agents in the treatment of comorbid depression in PwMS; systematic reviews confirm that treatment attrition for antidepressants among PwMS with depression is very high in clinical trials [11,12]. However, in meta-analyses, both cognitive behavioural therapy (CBT) and Mindfulness-based interventions (MBIs) have been found to be effective in treating symptoms of depression and have low attrition in PwMS [13–15]. Furthermore, previous work has shown that PwMS express a preference for behavioural programming for managing mood [16]. Indeed, both CBT and MBIs appear to be acceptable to PwMS. To optimize effects, tailoring is required [17,18]. Despite this, depression in PwMS is undertreated [6].

Based on evidence from the general population, an intervention that incorporates elements of both CBT and MBIs is likely to have incremental benefits for PwMS with comorbid depression. Indeed, Mindfulness-Based Cognitive Therapy (MBCT) was developed and evidenced as a preventative treatment for depressive relapse [19], building on well-established evidence for Mindfulness-Based Stress Reduction (MBSR) as a treatment for stress in people with chronic medical conditions, and CBT. In the general population, MBCT is recommended in best practice guidelines for those with mild-moderate depression, whereas those with severe depression should have individualised treatment [20]. MBCT is typically delivered as a group and includes psychoeducation, cognitive and behavioural tasks designed to raise awareness of and address putative key drivers of depression such as ruminative cognition and negative affect [19].

Besides strong evidence of effectiveness for MBCT in decreasing symptoms and recurrence of depression in the general population [21,22], including self-stigma [23], it also improves positive mental health outcomes such as QoL [24], mindfulness, self-compassion [25], positive affect and spirituality [26]. Indeed, among PwMS, the largest pooled effects in meta-analyses of MBIs are for improving mental health-related QoL [15]. Positive effects from MBCT have been shown to be stable for up to 2 years post treatment in people with depression – this is important because for a self-management intervention for PwMS (a chronic, progressive condition) to have a meaningful impact, effects have to be longitudinal and ideally incremental, meaning that with the right infrastructure and support, skills continue to develop and endure over time [27].

There are well documented inequities related to mental health care access faced by PwMS [28]. Online delivery of self-management resources helps to address some of these inequities by improving access for those PwMS who are more physically disabled, of lower social means, and living rurally, and hybrid (live and asynchronous) digital self-management interventions are more effective for people with chronic neurological disorders than those fully digitized [29]. Indeed, qualitative research synthesis suggests PwMS prefer a hybrid of live (synchronous) and asynchronous MBI offerings, which maximize acceptability and accessibility, respectively [17,18]. Community, camaraderie and common humanity are suggested as key factors which motivate sustained participation in MBIs for PwMS [18]. Those studies which have demonstrated long-term benefits from MBCT have highlighted the importance of ongoing access to learning materials beyond the standard 8-week course [30], and that for those people with depression, learning to use mindfulness is a longitudinal process which involves increased self-awareness, perspective-taking and acceptance, and requires continued practice to exert agency [31]. Taken together, implemented at scale, MBCT has the potential to help a large number of PwMS. However, there is a lack of evidence regarding the effectiveness of MBCT in PwMS with mild-to-moderate depression and there is a lack of understanding as to how to effectively implement this intervention for PwMS.

### Objectives

The overall aim of this project is to implement and evaluate online MBCT tailored for PwMS.

Specific objectives include:

1. Conduct a pragmatic, powered waitlist randomised controlled trial (RCT) of effectiveness of tailored online MBCT among PwMS who have case-ness of mild to moderate depression.
2. Evaluate implementation of the tailored online MBCT course for PwMS who have mild to moderate depression using the RE-AIM framework, informed by PRISM, through a parallel process evaluation.

## Methods

This type 1 hybrid effectiveness implementation study will employ mixed methods and comprises a powered RCT to evaluate the effectiveness of the tailored MBCT course, embedded qualitative interviews to explore participants’ experiences, and a process evaluation of the implementation of the course. Implementation will be examined using the RE-AIM (Reach, Effectiveness, Adoption, Implementation & Maintenance) framework, informed by PRISM (Practical, Robust Implementation and Sustainability Model), of participants in the course, and explores barriers and facilitators to implementation. This approach was chosen with the rationales of triangulation and expansion to examine convergence between quantitative and qualitative findings and to more comprehensively study the intervention, including factors influencing its use and implementation. This allows us to expand upon not only *whether* it is effective but also *how* the intervention is perceived and should be implemented.

The trial was registered on ClinicalTrials.Gov (Identifier: NCT07742579) on July 31, 2026. The trial will be conducted online through the University of Toronto. This protocol follows the Standard Protocol Items: Recommendations for Interventional Trials (SPIRIT) reporting guidelines for a RCT protocol.

### Eligibility

Inclusion criteria will include: 1. Age > / = 18; 2. Neurologist confirmed MS diagnosis (McDonald criteria [32]); 3. Able to understand spoken and written English; 4. A score of 14–28 on the Beck Depression Inventory-II (BDI-II) signifying mild-moderate depression [33]; 5. Registered with a primary care provider (family physician or neurologist), 6. Residing in Canada.

Exclusion criteria will include: 1. Potentially life-threatening comorbidities (suicidal ideation [i.e., score of 2 or 3 on the BDI-Fast Screen (BDI-FS) suicidal ideation question], history of psychosis, terminal/life threatening inter-current illness), or those such conditions expected to significantly limit participation and adherence (pregnancy, cognitive impairment, i.e., score <26 on the Montreal Cognitive Assessment – MoCA) [34], and 2. Receipt of MBCT in the past 2 years (completed at least 4/8 sessions).

### Recruitment and trial procedures

Study materials (e.g., study flyer) will be disseminated by NeuroSask Active and Connected, MS Canada, the Canadian Network of MS Clinics, and social media (e.g., MS groups on Facebook). Potential participants can reach out to the study team to indicate their interest and will undergo eligibility screening. Initial contact between participants and the research study team will always be by telephone, or videoconferencing software, where baseline eligibility screening will take place (Fig 1). Outstanding participant questions about the study can also be addressed at this point. Following this call, the research study team will send electronically a Participant Information Leaflet, consent forms, and baseline questionnaires to be completed via REDCap.

**Fig 1:**
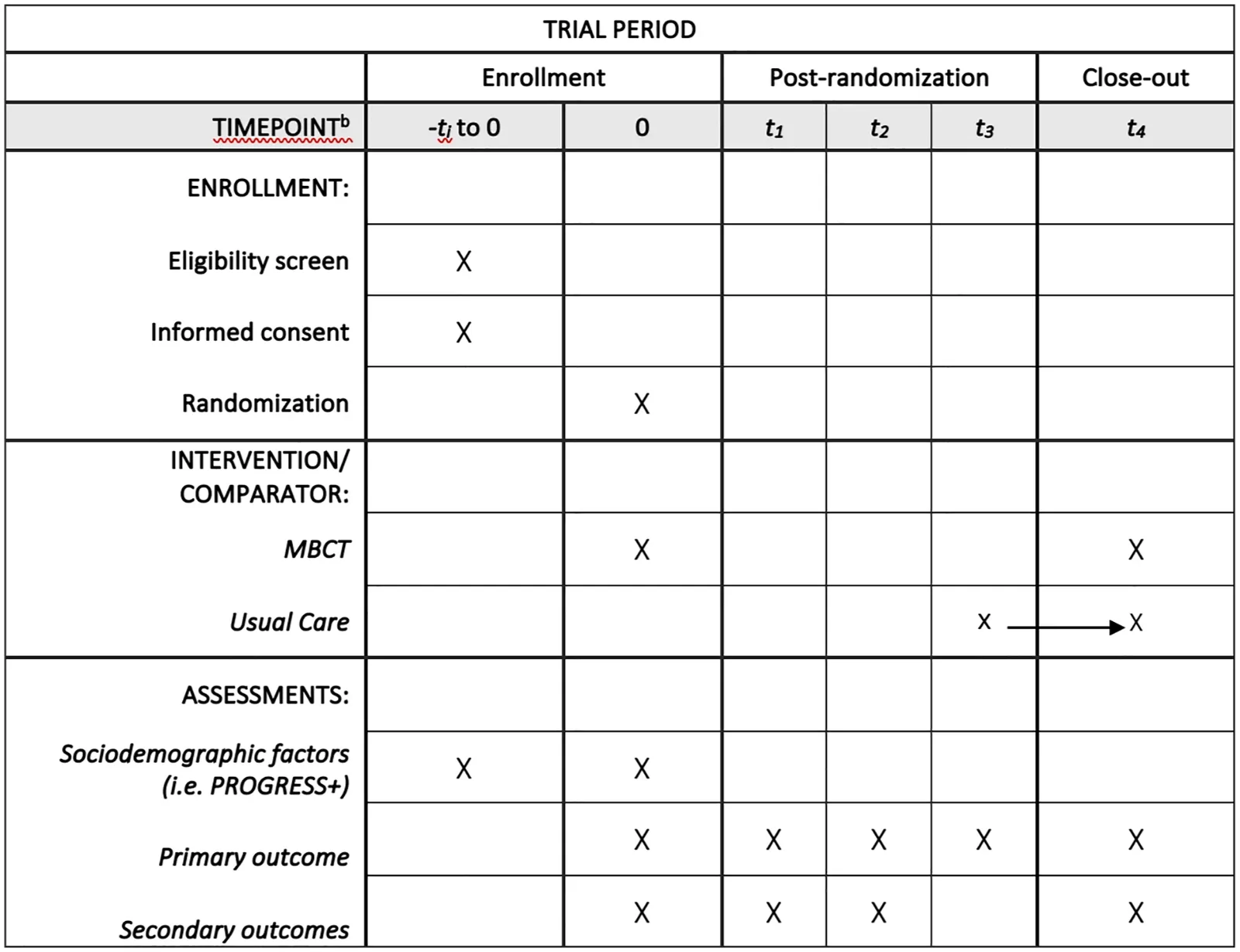
Participant timeline: schedule of enrollment, interventions, and assessments.

Randomization will be generated using a random computer-generated scheduler (e.g., SAS PROC PLAN) by a biostatistician who is unaware of participant identity and uploaded to REDCap software for the research coordinator to assign treatment allocation to recruited participants. As the design is a waitlist-controlled trial, randomization will be performed in clusters of 30 participants and will be allocated to Intervention or Waitlist Control arms in a 1:1 allocation in blocks of 2 (Fig 2). A waitlist control was selected as the comparator because there is currently no standard of care intervention for PwMS who have mild-moderate depression. Our pragmatic study design will thus compare MBCT to the care PwMS in Canada actually receive and will thus allow assessment of incremental benefit rather than efficacy.

**Fig 2:**
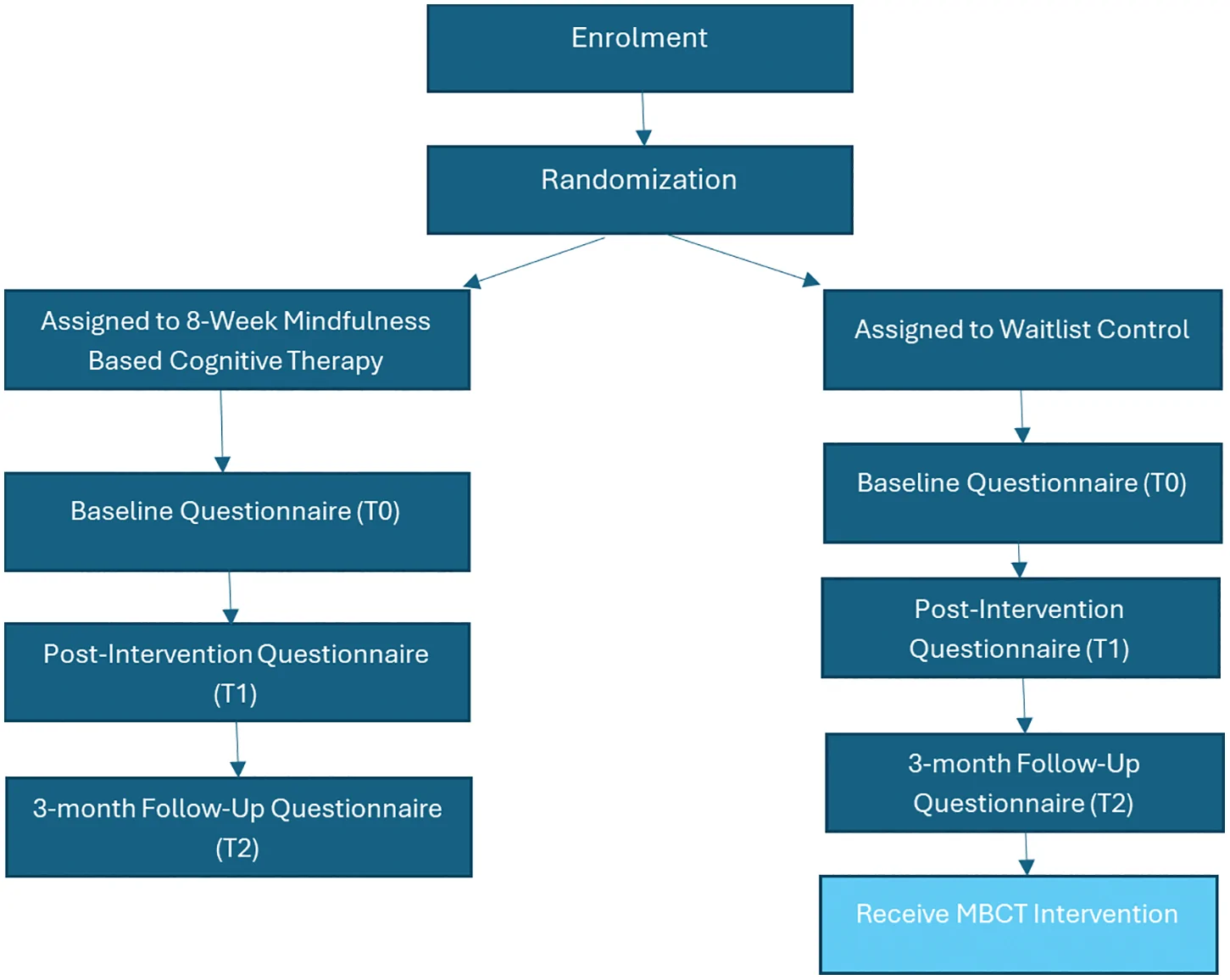
Participant Flow Chart.

Participant blinding in MBI research is challenging [35]. Pragmatic mitigation strategies in this study include [35]: 1) randomization will be generated via computerized randomization schedule generation and only the PI and statistician will have access to the schedule; 2) assessors and statistician blinded to group allocation for data entry, analysis, participant questionnaire data identifiable only by random study ID, group allocation concealed until final analysis; 3) publicly accessible study information kept general, no suggestion of intervention superiority; 4) participants advised only that they are allocated to an intervention ‘to improve emotional wellbeing’; 5) participants asked to not discuss group assignment with healthcare providers (HCPs) or study assessors for study duration.

### Intervention

The intervention will comprise online group MBCT, tailored for PwMS, delivered live, via secure video conferencing software, by trained MBCT instructors, with up to 15 PwMS per group, weekly 2.5 hour sessions (with comfort breaks) for 8 weeks, including core meditation practices (mindful-breath, -body, -movement awareness), psychoeducation on depression, reflective group discussion (‘enquiry’), and home practices of up to 45 minutes/day [36].

### Outcomes

The primary outcome is change in depression, as measured by HADS-D as a continuous measure. Between and within group differences in HADS-D will be evaluated. Clinically significant reductions in HADS-D will also be examined as an exploratory outcome (a reduction of 20% considered a ‘minimally important difference’ [MID] [37].

Secondary outcomes will be collected for exploratory analysis and can be found in Table 1. In all trial participants, trial outcomes will be collected remotely online using REDcap™, at baseline (T0), immediately post MBCT (T1), and 3 months later (T2) to assess magnitude and stability of treatment effects and/or hazards.

**Table 1:** Secondary outcomes analyzed.

Outcome	Measure
Trial Outcomes	
Self-efficacy	Generalized Self-Efficacy Scale (GSES) [38], which assesses self-beliefs around coping with a range of demanding life situations, and has strong internal consistency in PwMS
Self-management	Multiple-Sclerosis Self-Management Scale-Revised (MSSM-R), a multi-dimensional assessment of self-management knowledge and behaviour, which has strong internal consistency in PwMS [39]
Stigma	Stigma Scale for Chronic Illness-8 (SSCI-8), which measures enacted and internalized stigma, and has strong internal consistency in PwMS [40]
Fatigue	Modified Fatigue Impact Scale (MFIS), measuring how much cognitive, physical, and psychosocial aspects of fatigue impact on daily activities, higher scores indicating a greater impact. A 4-point reduction has been established as the MID in PwMS [41]. The MFIS has strong reliability, internal consistency and precision
Sleep	Insomnia Severity Index (ISI), measuring seven domains of sleep: difficulties initiating and staying asleep, early morning wakening, satisfaction, interference with daily function, noticeability, and distress. The ISI is recommended for use in PwMS [42], is reliable and valid [43], with suggested clinical cut-offs and MIDs [44]
Pain	Pain Effects Scale (PES), a 6-item measure which assesses the ways in which pain and unpleasant sensations interfere with mood, movement, sleep, work, recreation, and enjoyment of life. Higher scores indicate greater impact of pain on mood and behavior. The PES has good face validity and reliability [45]
Quality of life	Multiple Sclerosis Impact Scale-29 (MSIS-29) assesses the physical and psychological impact of MS on QoL. Based on the preferences of PwMS and common MS symptoms, it has good psychometric properties with high internal consistency and good test-retest reliability [46]
Anxiety	HADS-A, with a score > / = 8 demonstrating ‘clinically significant’ anxiety [47]. The HADS-A has high criterion-related validity and internal consistency reliability, with MID established in PwMS [48]
Mindfulness	The Five-facet Mindfulness Questionnaire (FFMQ) has good internal consistency, structural validity, and hypothesis testing ability [49]
Self-compassion	The Self-Compassion Scale-short form (SCS-sf), a 12-item, six-factor scale that assesses the ability to ‘hold one’s feelings of suffering with a sense of warmth, connection and concern’ [50]. It has adequate internal consistency, and correlates highly with the full-length Self-Compassion Scale
Emotion regulation	The Difficulties with Emotional Regulation Scale short-form (DERS-sf) assesses emotion regulation over six domains: awareness, clarity, goals, non-acceptance, impulse control during negative affect, ability to access effective emotion regulation strategies [51]. It has strong internal consistency, overall and individual (goals) factors predictive of clinical severity and treatment outcome post-CBT
Depressive symptoms	Beck Depression Inventory II (BDI-II), which has been shown to be valid in PwMS [52] and with scores falling between 14–28 indicating mild-moderate depression [33]
Group processes (only those allocated to tailored online MBCT)	Group attendance, climate (Climate Questionnaire-Engage Scale), and therapeutic alliance (Working Alliance Inventory)

Implementation outcomes will be collected throughout the study and formally assessed and reported following 12 months completion of the trial. These outcomes are described in Table 2.

**Table 2:** Implementation outcomes analyzed.

RE-AIM Domain	Quantitative Metrics	Qualitative Data
Reach	Number eligible, approached, enrolled, participation rate, representativeness, equity indicators	Participant and non-participant experiences with recruitment and access
Effectiveness	Primary and secondary clinical outcomes, PROMs/PREMs, adverse events, quality of life	Participant experiences, perceived benefits and challenges, mechanisms of change
Adoption	Number and proportion of clinicians/sites participating, provider characteristics, intervention adherence	Perceived fit, acceptability, organizational readiness, leadership support
Implementation	Fidelity, dose delivered and received, intervention adaptations, attendance, completion, feasibility, implementation costs	Experiences delivering and receiving the intervention, workflow integration, barriers and facilitators, adaptations
Maintenance	Sustained participant outcomes, continued intervention use, intention to continue delivery	Perceived sustainability, organizational support, recommendations for future implementation

### Data collection

Participants will be asked to fill out an online questionnaire at baseline (0 weeks), post-intervention (8 weeks) and follow-up (21 weeks). Online questionnaires will be used to collect data on the outcomes noted above and socio-demographic variables including age, sex, gender identity, race/ethnicity, current living situation, marital status, household income bracket, educational attainment, disease duration, disability level, number/type comorbid conditions, disease modifying therapy (DMT) and/or psychotropic use/type/dose.

Further, a subset of participants (n = 30–40) will be invited to participate in one semi-structured interview via video-conferencing software examining motivations for participation, acceptability, accessibility, perceived effects of the MBCT program, as well as explore barriers and facilitators to implementation. HCPs, MBCT instructors and MS decision makers (e.g., clinic directors) will also be invited to participate in one semi-structured interview regarding their thoughts on the barriers and facilitators to implementation of MBCT for PwMS. Interview data will be digitally recorded and transcribed verbatim.

### Analysis

The sample size was based on 3-month follow up data from Bogosian et al.[53] whereby, assuming the HADS-D scores are normally distributed, we expect to see a difference between the tailored online MBCT group and the usual care group of 2 units on average, with a standard deviation (SD) of 3.5, or a difference of 0.57 per SD. A minimum sample of 152 will provide a linear regression model with 3 time points (T0-T2) spaced at baseline (0 weeks), post-intervention (8 weeks) and follow-up (21 weeks) with 80% power. Sample size is adjusted for an assumed intracluster correlation of 0.2 of the repeated measures and assumed moderate baseline adjustment correction of 0.4 and 16% loss to follow-up for the analysis of our primary outcome, HADS-D based upon previous trial attrition rates. Sample size was calculated using SAS 9.4 for Windows (SAS Institute, North Carolina, USA).

All statistical analyses will be undertaken by blinded staff (biostatistician). A statistical analysis plan will be completed and signed off prior to database lock. Demographic baseline characteristics will be summarized by allocated group using means and SDs for continuous variables if normally distributed and counts and percentages for categorical ones. Primary outcome of HADS-D will be analyzed comparing baseline, post-intervention and follow-up between study arms using a linear mixed effects model (LMM) with a group, time, and group by time interaction term. Secondary linear mixed models will be run adjusting for demographic baseline covariates following variable selection and shrinkage algorithm (i.e., LASSO) and compared to the unadjusted models. All covariates will be assessed for multicollinearity (tolerance statistic <0.4) and the most clinically relevant variable of correlated covariates will be included in the model. As there will be multiple iterations of tailored online group MBCT, within group dependencies may influence observed effects [54]. As such, subjects will be clustered within groups as random effect in LMM. Potential cluster effects [55] will be explored by examining between group means, and baseline-follow-up measures using one-way ANCOVA. Exploratory subgroup analyses on factors such as age, sex, gender identity, ethnicity will be undertaken and will also explore differential impact of MBCT between those with depression and those with depression plus anxiety (HADS-A > / = 8). An intention to treat (ITT) analysis will be followed with all randomized participants included in analysis. No interim analyses will be undertaken. Missing outcome data and nature of missingness will be assessed. If less than 5% missing data and missing completely at random (MCAR), whole case analysis will be conducted. Assumed baseline data missingness will be < 5% and MCAR due to REDCap data collection and as such whole case analysis will be appropriate. Should baseline data to be included in ANCOVA models exceed 5% missing, missing baseline data will be imputed using multiple imputation methods [56] via five imputed datasets and model results averaged across the five iterations. A clinical prediction model will identify those PwMS with depression most likely to benefit from tailored online MBCT in which the binary outcome is considered a 20% reduction in HADS-D. This will involve a logistic regression model on the group receiving online MBCT to examine the impact of potential predictors (e.g., sex, gender, race, ethnicity, MS phenotype, disability level, DMTs, psychotropics) following variable selection and shrinkage algorithm. Predictors will be assessed for multicollinearity and sparsity. Bootstrap validation will be used to internally validate the prediction model’s performance. This will involve 10,000 samples of the same size as the original dataset chosen with replacement. The results over the 10,000 simulations will be examined for which predictors remain statistically significant and sufficiently meaningful (in > 50% of simulations). Adherence and attrition rates will be compared between treatment arms. False discovery rate will control for multiplicity of testing.

An inductive thematic analysis, as described by Braun and Clarke [57], will be employed initially to interview transcripts, but we will also subsequently perform deductive analyses using the Consolidated Framework for Implementation Research to explore barriers and facilitators to delivering and implementing tailored online group MBCT to PwMS with comorbid depression.

Qualitative and quantitative results will first be analyzed individually and then will be merged for the generation of meta-inferences. Findings will be merged using a joint display to examine fit of integration in terms of explanation, discordance, and concordance. Meta-inferences will be generated through examination of the joint display regarding effectiveness and assessment of the implementation of the intervention [58].

### Monitoring

No adverse events are anticipated from online MBCT, with participants expected to benefit [59]. Online MBCT is considered safe [60]. Participant safety will be monitored continuously by the trial steering committee (TSC) and an independent data safety monitoring committee (IDMC). The trial steering committee will include a neuropsychiatrist, neurologist, physiatrist, neuropsychologist, biostatistician, and PwMS knowledge users. Trial stopping criteria will be any overt evidence of harm arising from online MBCT. A suicidality standard operating procedure was developed and approved by the sponsor, whereby any participant expressing suicidal ideation will be referred for urgent assessment by their primary care physician (family physician or neurologist) and/or in their local emergency department.

### Ethics

This study has received ethics approval from the University of Toronto Research Ethics Board (#49655) on February 26, 2026. Informed written consent will be obtained from all participants by study personnel prior to trial procedures and participants will be alerted if any protocol changes occur. Only pooled de-identified quantitative results will be presented/qualitative results reported using study IDs only.

### Dissemination

Study results will be disseminated through traditional knowledge mobilization strategies, including publication in peer-reviewed journals (e.g., Multiple Sclerosis and Related Disorders) and presentations at appropriate conferences and scientific meetings (e.g., Americas Committee for Treatment and Research in Multiple Sclerosis [ACTRIMS] Forum). Further, findings will be disseminated through the Canadian MS Rehabilitation Knowledge Mobilization Network and other MS organizational networks (e.g., NeuroSask Active and Connected). Less traditional, novel and creative strategies, including an integrated knowledge translation (iKT) approach, will also be employed to maximize uptake by people and organizations serving minority and marginalized PwMS. IKT, per the Canadian Institute of Health Research, is “a way of doing research that involves decision makers/knowledge-users in all stages of the research process” [61].

### Study Timeline

Participant recruitment has not yet begun but is expected to begin November 1, 2026. Participant recruitment is expected to be completed in October 2027. Data collection is expected to be completed in March 2028. Results are expected in spring 2028.

## Discussion/Conclusion

Depression is the most common comorbidity affecting PwMS. Currently, access to effective treatment is limited. Evidence for pharmacological treatment strategies is limited and PwMS express a preference for behavioural programming in this context. Research on the effectiveness and implementability of non-pharmacological interventions for the treatment of PwMS with comorbid depression is thus urgently needed. The primary purpose for this study is to implement and evaluate a tailored online MBCT programme for PwMS with depression. This will be done through a powered type 1 hybrid effectiveness-implementation RCT examining MBCT compared with a waitlist control group. This study will involve both qualitative and quantitative methods to examine whether MBCT is effective in improving depression in PwMS, whether it is acceptable to PwMS, how to implement and scale up the intervention. By using mixed methods, our study will provide novel insights into not only the effectiveness of MBCT in PwMS with depression but also how to tailor and implement it, providing a practical solution to a glaring gap in care. Using qualitative methods provides a meaningful platform for participants to share their own unique experiences with tailored online group MBCT, can help in the interpretation of trial findings (i.e., reasons for adherence, attrition), provides explanations for any change in health behaviors [62] during and following tailored online group MBCT, informs any further required intervention tailoring for future iterations, and provides much-needed insight into how an MBI is received by diverse PwMS. Guided by implementation theories, models, and frameworks (i.e., RE-AIM, PRISM) and integrating qualitative and quantitative findings, our study will comprehensively examine the effectiveness and implementation of MBCT for PwMS.

This study will provide valuable outputs for the treatment of depression in PwMS. Currently, there is a lack of information in whether MBCT is effective in PwMS with comorbid depression. Further, providing information on how to iterate and implement these interventions online can ensure a treatment that is not only effective but practical in a real-world setting where PwMS increasingly access care virtually and where effective self-management complements high-quality clinical care programming.

*Trial Registration:* This trial was registered on ClinicalTrials.gov (NCT07742579) on July 31, 2026 (https://clinicaltrials.gov/study/NCT07742579?term=NCT07742579&viewType=Card&rank=1).
